# Rapidly measured indicators of recreational water quality and swimming-associated illness at marine beaches: a prospective cohort study

**DOI:** 10.1186/1476-069X-9-66

**Published:** 2010-10-31

**Authors:** Timothy J Wade, Elizabeth Sams, Kristen P Brenner, Richard Haugland, Eunice Chern, Michael Beach, Larry Wymer, Clifford C Rankin, David Love, Quanlin Li, Rachel Noble, Alfred P Dufour

**Affiliations:** 1 United States Environmental Protection Agency, National Health and Environmental Effects Research Laboratory, Research Triangle Park, North Carolina, USA; 2 United States Environmental Protection Agency, National Exposure Research Laboratory, Cincinnati, Ohio, USA; 3 Centers for Disease Control and Prevention, Division of Foodborne, Waterborne and Environmental Diseases, Atlanta, Georgia, USA; 4 Johns Hopkins University, Baltimore, Maryland, USA; 5 University of North Carolina at Chapel Hill, Chapel Hill, North Carolina, USA

## Abstract

**Introduction:**

In the United States and elsewhere, recreational water quality is monitored for fecal indicator bacteria to help prevent swimming-associated illnesses. Standard methods to measure these bacteria take at least 24 hours to obtain results. Molecular approaches such as quantitative polymerase chain reaction (qPCR) can estimate these bacteria faster, in under 3 hours. Previously, we demonstrated that measurements of the fecal indicator bacteria *Enterococcus *using qPCR were associated with gastrointestinal (GI) illness among swimmers at freshwater beaches. In this paper, we report on results from three marine beach sites.

**Methods:**

We interviewed beach-goers and collected water samples at marine beaches affected by treated sewage discharges in Mississippi in 2005, and Rhode Island and Alabama in 2007. Ten to twelve days later, we obtained information about gastrointestinal, respiratory, eye, ear and skin symptoms by telephone. We tested water samples for fecal indicator organisms using qPCR and other methods.

**Results:**

We enrolled 6,350 beach-goers. The occurrence of GI illness among swimmers was associated with a log_10_-increase in exposure to qPCR-determined estimates of fecal indicator organisms in the genus *Enterococcus *(AOR = 2.6, 95% CI 1.3-5.1) and order *Bacteroidales *(AOR = 1.9, 95% CI 1.3-2.9). Estimates of organisms related to *Clostridium perfringens *and a subgroup of organisms in the genus *Bacteroides *were also determined by qPCR in 2007, as was F+ coliphage, but relationships between these indicators and illness were not statistically significant.

**Conclusions:**

This study provides the first evidence of a relationship between gastrointestinal illness and estimates of fecal indicator organisms determined by qPCR at marine beaches.

## Background

It is usually impractical to test recreational waters directly for the many and diverse pathogenic microorganisms associated with human derived sewage. As a result, recreational waters are often monitored for fecal indicator bacteria. Fecal indicator bacteria such as *Enterococcus *spp. or *Escherichia coli *are ordinarily harmless microbes that are commonly found in sewage and other sources of fecal contamination [1]. These fecal indicator bacteria have been statistically associated with gastrointestinal (GI) illness in recreational waters [2,3]. Standard methods for measuring water quality involve growing fecal indicator bacteria in culture which requires at least 24 hours and may result in incorrect assessments of water quality by beach managers and regulators [4]. Molecular methods, such as quantitative polymerase chain reaction (qPCR), have the ability to detect fecal indicator organisms much faster by targeting and measuring specific genetic markers [5]. We previously demonstrated that the fecal indicator bacteria *Enterococcus *spp. estimated by qPCR was well-associated with gastrointestinal illness among swimmers at freshwater beaches [6,7].

Previous studies found different associations between fecal indicator bacteria measured by culture and swimming-associated illness in marine and fresh waters [8,9], possibly due to differences in the fate and transport of indicator and pathogenic microorganisms [8,10,11]. In this report, we extend our research to three marine beaches in the continental United States where we examine the relationships between swimming-associated illnesses and fecal indicator bacteria determined by alternative rapid methods.

## Methods

### Beach sites and health survey

**Figure 1:**
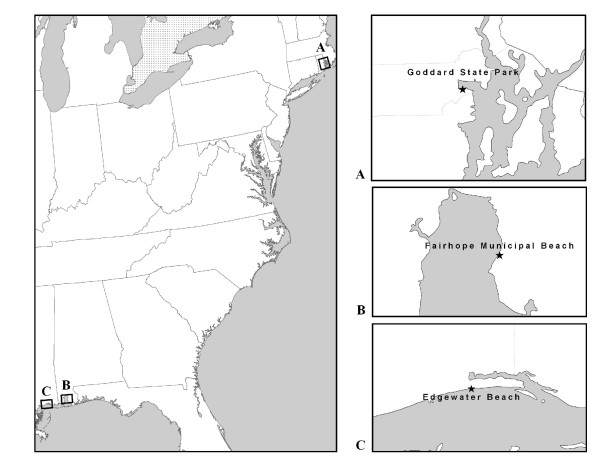
**Marine beach sites**.

We conducted studies at three marine beaches affected by treated sewage discharge from nearby Publicly Owned Treatment Works (POTW). In 2005, we studied Edgewater Beach in Biloxi, Mississippi, and in 2007 we studied Goddard Beach in Goddard Memorial State Park in West Warwick, Rhode Island and Fairhope Municipal Beach in Fairhope, Alabama (Figure 1). Each beach site was located within 7 miles or less of a treated sewage discharge outfall from facilities that served populations of at least 15,000. Based on historical records, each beach site showed variability in water quality, but were generally in compliance with local and federal water quality guidelines.

Data collection procedures for the health survey have been described previously [6,7]. In brief, we conducted surveys on weekends and holidays between May and September. Upon arrival beachgoers were provided a pamphlet describing the study. After reviewing the pamphlet, interviewers approached the household group or individual and offered them the opportunity to enroll in the study. On most days, all beachgoers arriving between approximately 11 AM and 4 PM were offered enrollment. Respondents were ineligible if they had completed the study in the previous 30 days, or if there was no adult (18 years of age or older) household member present. Households provided verbal consent and completed an enrollment questionnaire consisting of demographic information, swimming exposures in the previous two weeks, and the presence of underlying health conditions, such as chronic diarrhea, asthma, or skin conditions. As they left the beach for the day, participants completed a questionnaire to ascertain the extent and duration of their contact with water and other activities during their visit to the beach such as contact with sand and food consumption. Ten to twelve days following the beach visit, we telephoned participants and asked about the occurrence of new gastrointestinal, skin, respiratory, eye, or ear symptoms.

The study procedures, questionnaires, protocols and consent process were reviewed and approved by the Institutional Review Board of the Centers for Disease Control and Prevention.

### Illness definitions

We considered the following health endpoints consistent with those we previously reported [6,7].

**"Gastrointestinal illness" (GI illness) **was defined as any of the following: (1) diarrhea (three or more loose stools in a 24-hour period); (2) vomiting; (3) nausea and stomachache; (4) nausea or stomachache, and interference with regular activities (missed regular activities as a result of the illness).

**"Upper respiratory illness" (URI) **was defined as any 2 of the following: sore throat, cough, runny nose, cold, or fever.

**"Rash" **was defined as a rash or itchy skin.

**"Eye irritations" **were defined as either eye infection or watery eye.

**"Earache" **was defined as earache, ear infection, or runny ears.

Diarrhea was also considered as a stand alone outcome because it is a commonly used definition of gastroenteritis in population-based surveillance [12,13].

Participants ill within 3 days before their beach visit were excluded from analysis of the health outcome related to their baseline symptoms.

### Water sample collection and analysis

Protocols used for water sample collection have been described [5]. Briefly, we collected two 1-liter water samples at 8:00 AM, 11:00 AM, and 3:00 PM along 3 transects perpendicular to the shoreline. At each transect, we collected one sample in waist-high water (1 m deep) and one in shin-high water (0.3 m deep). Transects were located at least 60 m apart and encompassed the swimming area. Following collection, samples were placed in coolers and maintained on ice at 1 to 4°C. At each water sampling time we recorded environmental conditions, including air and water temperature, cloud cover, rainfall, wind speed and direction, wave height, number of people (on the beach and in the water), boats, animals (number and type, on the beach and in the water), tide stage, and debris.

Water samples were tested for total *Enterococcus *spp. [5] and total *Bacteroidales *spp. [14], hereafter referred to as *Enterococcus *and *Bacteroidales *respectively, by qPCR using previously published protocols [5,14]. In 2007, we added qPCR tests for subgroups of *Bacteroides *[15], and *Clostridium *spp. or "*Clostridium perfringens *group" [16], hereafter referred to as "*fecal Bacteroides*" and "*Clostridium*", respectively. In 2007, we also included a novel, faster test for F+ (male-specific) coliphage based on a culture and latex agglutination assay (CLAT assay), which also distinguishes F+ RNA coliphage and F+ DNA coliphage [17]. F+ coliphage was also evaluated using a 24-hour spot test according to EPA Method 1601 [18]. Samples were also tested for *Enterococcus *spp. using EPA Method 1600 [19], a culture-based method. Results for *Enterococcus *measured by EPA Method 1600 are reported in colony forming units (CFU) per 100 ml sample and for F+ coliphage in most probable number (MPN) per 100 ml. Samples for EPA Method 1600 and qPCR analysis were filtered within 6 hours of collection. The filters were held at -20°C and shipped overnight to EMSL Analytical (Westmount NJ) on dry ice where DNA extraction and qPCR analyses for *Enterococcus*, *Bacteroidales *and fecal *Bacteroides *were conducted. Frozen DNA extracts were sent from EMSL Analytical to the US EPA in Cincinnati where qPCR analysis was conducted for *Clostridium*.

Primer and probe sequences used for the *Enterococcus *[5], *Bacteroidales *[14] and fecal *Bacteroides *[15] qPCR assays were described previously. Primers used for the amplification of *Clostridium *were those of the "Clostridium perfringens group" assay targeting about 34 Clostridium species as reported by Rinttilä et. al. [16]. Additional details regarding sample processing, DNA extraction and reaction conditions for qPCR analyses, are provided in additional file 1. In brief, following filtration, DNA was extracted, and polymerase chain reaction (PCR) amplification was carried out using the TaqMan PCR product detection system. The reactions were performed in a thermal cycling instrument (Smart-Cycler System, Cepheid, Sunnyvale, CA) except for *Clostridium *which was performed on a Model 7900 DNA thermal cycler (Applied Biosystems, Foster City, CA). Both instruments automated the detection and quantitative measurement of the fluorescent signals produced by TaqMan probe degradation during each cycle of amplification.

PCR cycle threshold (CT) measurements of the test sample DNA extracts were compared with those of similarly prepared extracts from calibrator samples containing a known quantity of the target organism cells. Ratios of the target sequences in the test and calibrator samples were converted to estimates of calibrator cell equivalents (CCE) in the test samples [20]. PCR measurements of salmon testes DNA, that was added to the extraction buffer as a source of reference target sequences, were used to estimate the relative efficiency of total DNA recovery from the water sample filters compared to the calibrator samples and to identify potential PCR inhibition [21]. Five-fold dilutions of the water filter and calibration sample extracts were analyzed and water filter extracts giving salmon DNA assay CT values that were > 3 CT units higher than the mean values from the calibration extracts were reanalyzed after additional 5-fold dilutions. If both dilutions failed the salmon CT criterion, the sample was excluded and results were replaced with mean of valid samples collected at the same location, depth and time. Salmon DNA assays were performed in separate reaction tubes.

### Quantitation of fecal indicator bacteria by qPCR

Two basic approaches were used to quantify CCE: "delta delta-CT" (CCE_ΔΔ _) [5-7,14], and "delta-CT" (CCE) [14]. CCE values were determined using only test sample and batch-mean calibration sample target organism assay CT values (CCE_Δ_) and also after corrections using CT values from the salmon reference assays (CCE_ΔΔ _method). See previously published manuscripts [5,14,22] and additional file 1 for a discussion and description of these calculations. The CCE_ΔΔ _calculation provides quantitative adjustment for partial inhibition [5,22], but there is some evidence that the salmon reference assay may over correct the CCE quantitation due to a higher sensitivity to matrix inhibitory effects whereas CCE_Δ _may lead to underestimations [14]. Therefore, both calculation methods were used to determine whether health effects associations were substantially affected by the calculation approach. Additional details on the CCE calculations are provided in the additional file 1.

The lower detection limit was defined as the upper 95% CT bound of the Y-intercept from the pooled standard curve data that was generated from repeated analyses of serially diluted genomic DNA extracts from the calibrator bacterial strains during the study period. Target sequence concentrations in these genomic DNA extracts were determined as previously described [22]. CT values were restricted at this upper bound for all CCE calculations. One-half the calculated CCE was used for non-detects where there was no detection after 45 cycles. Results are reported in qPCR CCE per 100 ml of original sample.

### Swimming exposure

Our primary definition of swimming was "body immersion", defined as immersion to the waist or higher. Previously, we observed similar risks of illness for those who immersed their body and those who immersed their head [7]. Non-swimmers were those who reported no water contact.

We used the mean of the log_10 _fecal indicator organism estimates to represent exposure. We created separate exposure indices based on all samples (daily average), representing an estimate of the overall water quality, and for morning samples (8:00 AM, 11:00 AM) to evaluate whether morning water quality measures were associated with illness. For categorical presentations, indicator groups were established according to quintiles (for indicator bacteria), presence/absence (F+coliphage by CLAT) or at the median (F+ coliphage by SPOT). Non-swimmers were considered unexposed to waterborne fecal indicator organisms.

### Statistical analysis

We used logistic regression models to quantify and describe the relationship between estimates of fecal indicator organisms and the risk of illness among swimmers. The predictor of interest was the estimate of fecal indicator organisms. Factor variables representing "beach" were included in all models to control for differences in baseline illness. Robust estimates of variance were used to account for non-independence of observations within households [23-26]. Covariates which could plausibly affect the relationship between water quality and illness, or those which were associated with health outcomes were considered for inclusion in regression models. These included age, sex, race, contact with animals, other swimming in the past 1-week, contact with other persons with diarrhea, distance traveled to the beach, frequency of visits to the beach under study, any other chronic illnesses (GI, skin, asthma), digging in sand, use of insect repellent and sunscreen, and consumption of raw or undercooked meat. We accounted for the following environmental measures: precipitation since 3:00 PM the previous day, bather density, dogs, birds and other animals on the beach, air and water temperature, wind speed, tide stage, wave height, cloud cover, wind direction and boat density, which were often highly correlated, by reducing them to summary measures using principal components analysis. The first two principal components explained 53% of the variability and were characterized by air and water temperature (first component), and wave height, windspeed and precipitation (second component). These two components were included as covariates in regression models. When one or more of the environmental measurements were missing, we used best-subset regression to impute the principal components [27].

For each analysis, the set of covariates was reduced through a change-in-estimate procedure [28], where the parameter of interest was the regression coefficient for the fecal indicator organism, with a criterion of a 5% change in the coefficient. To evaluate differences in the indicator-illness relationship across beaches and age groups, we used likelihood ratio tests to compare full models with multiplicative interaction terms between beach or age group and water quality (which allowed slopes to differ) to restricted models constrained to a single slope. When data were sufficient, we conducted separate analysis for those 10 years of age and under, consistent with our previous report [6]. Stratified analyses for older age groups were not conducted due to small sample size, and infrequent swimming and reporting of illness.

Adjusted Odds Ratios (AORs) estimated from logistic regression models were used to represent the degree of association between fecal indicator estimates and risk of illness. An AOR of 1 indicates no association or a completely flat slope. AORs with a 95% confidence bound including 1 were considered not statistically significant. For graphical presentations, adjusted probabilities of illness were predicted from logistic regression models holding covariates constant at their mean value.

We used Stata version 10.1 for data analysis [29].

## Results

### Subject recruitment and respondent characteristics

We conducted interviews on 70 study days at the three beaches (Table 1). At Edgewater Beach, data collection was stopped several days early due to the effects of Hurricane Katrina. A total of 9,069 beach-goers were offered enrollment. Of these, 1,715 (19%) refused to participate or were ineligible. Of those who agreed to participate, 6,350 (78%) completed the telephone interview and were eligible for analysis. Selected characteristics of the study population are shown in Table 1. Seventy-five percent of children age 5-10 immersed their body compared to only 26% of those over 55 (data not shown). Swimming was also associated with male gender, non-white race, less frequent visits to the beach, the absence of chronic illnesses and less frequent consumption of raw or undercooked meat (data not shown).

**Table 1:** Enrollment and selected respondent characteristics

	Edgewater Beach	Fairhope Beach	Goddard Beach	Total
	**N(%)**	**N(%)**	**N(%)**	**N(%)**

**Days of Study**				
Total	21	25	24	70

**Interviews**				
Total	1351	2022	2977	6350

**Age**				
0-4	75(5.7)	241(11.9)	238(8.1)	554(8.8)

5-11	157(11.8)	377(18.7)	347(11.8)	881(14)

12-19	201(15.2)	210(10.4)	242(8.2)	653(10.4)

20-34	453(34.2)	454(22.5)	737(25.1)	1644(26.2)

35 and over	439(33.1)	739(36.6)	1375(46.8)	2553(40.6)

Total	1325(100)	2021(100)	2939(100)	6285(100)

**Race**				
Non-white	551(40.8)	702(34.7)	1090(36.7)	2343(36.9)

White	798(59.2)	1320(65.3)	1882(63.3)	4000(63.1)

Total	1349(100)	2022(100)	2972(100)	6343(100)

**Sex**				
Male	665(49.6)	855(42.3)	1285(43.2)	2805(44.3)

Female	676(50.4)	1167(57.7)	1689(56.8)	3532(55.7)

Total	1341(100)	2022(100)	2974(100)	6337(100)

**Immersed body in water**				
No	605(44.9)	1193(59.2)	1889(63.6)	3687(58.2)

Yes	741(55.1)	823(40.8)	1080(36.4)	2644(41.8)

Total	1346(100)	2016(100)	2969(100)	6331(100)

**Immersed head in water**				
No	915(68.1)	1370(68)	2189(73.8)	4474(70.7)

Yes	429(31.9)	646(32)	779(26.2)	1854(29.3)

Total	1344(100)	2016(100)	2968(100)	6328(100)

### Water quality

We collected and tested a total of 1,242 water samples on study days (Table 2). In terms of overall water quality, Fairhope Beach had the highest geometric mean *Enterococcus *CFU (21 CFU/100 ml) and Goddard Beach the lowest (4 CFU/100 ml). Individual samples of *Enterococcus *CFU ranged from below detection to 3,000 CFU/100 ml. Overall, 142 samples (11.5%) exceeded 104 CFU/100 ml, the EPA recommended single sample maximum for marine beaches [30]. Individual samples most frequently exceeded the single sample maximum at Fairhope Beach (99 samples, 23%), followed by Edgewater Beach (32, 8.5%) and Goddard Beach (11 samples, 3%). *Enterococcus *CCE estimated by qPCR were higher than *Enterococcus *CFU. For all indicators measured by qPCR, estimates of CCE_ΔΔ _were higher than CCE_Δ_. *Bacteroidales *CCE were highest among the indicators.

**Table 2:** Fecal indicator bacteria estimates at marine beaches

Indicator	**N**^**1**^	**Min**.	**Max**.	Geometric Mean	Non-detects
**Edgewater Beach**					
*Enterococcus *CFU	377	0.1	920	7.2	48

*Enterococcus *CCE_ΔΔ_	378	69	10000	380	0

*Enterococcus *CCE_Δ_	378	54	3600	150	0

*Bacteroidales *CCE_ΔΔ_	378	120	430000	3000	4

*Bacteroidales *CCE_Δ_	378	47	300000	1300	4

**Fairhope Beach**					
*Enterococcus *CFU	431	0.1	3000	21	36

*Enterococcus *CCE_ΔΔ_	438	19	99000	260	97

*Enterococcus *CCE_Δ_	438	26	29000	130	97

*Bacteroidales *CCE_ΔΔ_	432	8.5	200000	1800	28

*Bacteroidales *CCE_Δ_	432	10	140000	980	28

*Clostridia *CCE_ΔΔ_	438	79	20000	1200	1

*Clostridium *CCE_Δ_	438	44	7900	650	1

Fecal *Bacteroides *CCE_ΔΔ_	408	16	37000	450	85

Fecal *Bacteroides *CCE_Δ_	408	9.8	25000	210	85

**Goddard Beach**					
*Enterococcus *CFU	426	0.1	960	3.6	78

*Enterococcus *CCE_ΔΔ_	425	14	26000	160	28

*Enterococcus *CCE_Δ_	425	13	12000	120	28

*Bacteroidales *CCE_ΔΔ_	426	18	120000	1100	32

*Bacteroidales *CCE_Δ_	426	23	65000	900	32

*Clostridia *CCE_ΔΔ_	420	9.6	52000	890	0

*Clostridium *CCE_Δ_	420	29	17000	500	0

Fecal *Bacteroides *CCE_ΔΔ_	426	11	140000	530	99

Fecal *Bacteroides *CCE_Δ_	426	11	66000	410	99

1: Number of samples

CCE = qPCR Calibrator Cell Equivalents per 100 ml for the delta CCE_Δ _and delta-delta CCE_ΔΔ _CT calculations CFU = Colony Forming Units per 100 ml (EPA Method 1600)

Fifty-six percent (100/222) of samples at Fairhope Beach and 65% (203/425) of samples at Goddard Beach were positive for F+ coliphage by the 24 hour SPOT test. Fewer samples were positive for F+ coliphage by the CLAT assay. At Fairhope Beach, 4% (8/228) and 14% (14/224) of samples indicated the presence of F+ RNA and F+ DNA coliphage, respectively. At Goddard Beach, 8% (31/425) and 9% (37/423) of samples tested indicated the presence of F+ RNA and F+ DNA coliphage, respectively.

### Illness and water quality

#### Bacterial indicators of water quality and illness

The number and crude (unadjusted) percentage of respondents reporting illness among non-swimmers and swimmers for quintiles of exposure based on daily averages are shown for all subjects in Table 3 for qPCR CCE_ΔΔ _and for *Enterococcus *CFU. For *Enterococcus *CCE, *Bacteroidales *CCE and *Enterococcus *CFU, the crude cumulative incidence of both GI illness and diarrhea increased with increasing levels of exposure and peaked in the highest exposure categories. On days when the daily geometric mean of *Bacteroidales *CCE_ΔΔ _was highest (over 3,530 CCE/100 ml), approximately 12% of swimmers reported GI illness compared to 6% among non-swimmers and 4% among swimmers on days when *Bacteroidales *CCE_ΔΔ _was low (< 542 CCE/100 ml). Similar patterns were noted for *Enterococcus *CFU and CCE.

**Table 3:** Illness among non-swimmers and swimmers by quintiles of daily average indicator exposures

	GI		Diarrhea		URI		Rash		Earache		Eye	
						
	N	%	N	%	N	%	N	%	N	%	N	%
*Enterococcus ${\text{CCE}}_{\Delta \Delta }^{1}$*												
Non-swimmer	159	5.86	100	3.69	109	4.07	68	2.49	35	1.26	86	3.07

56.8,125	31	5.21	22	3.70	33	5.60	29	4.82	12	1.96	23	3.75

125,170	30	6.90	20	4.60	17	3.90	15	3.45	6	1.36	8	1.79

170,268	44	8.22	21	3.93	31	5.88	18	3.30	13	2.35	12	2.17

268,396	50	10.35	28	5.81	26	5.42	18	3.68	7	1.41	15	2.99

396,1.42e+03	52	10.26	45	8.88	36	7.45	20	3.97	9	1.78	14	2.73

*Bacteroidales *${\text{CCE}}_{\Delta \Delta }^{1}$												
Non-swimmer	159	5.86	100	3.69	109	4.07	68	2.49	35	1.26	86	3.07

66.5,542	22	4.32	17	3.34	33	6.57	28	5.35	12	2.27	18	3.41

542,1.43e+03	36	6.53	24	4.36	23	4.23	20	3.59	10	1.77	11	1.93

1.43e+03,1.97e+03	33	7.55	16	3.66	22	5.09	11	2.51	6	1.35	10	2.24

1.97e+03,3.53e+03	55	10.34	37	6.97	38	7.18	17	3.20	12	2.24	14	2.56

3.53e+03,1.28e+04	61	11.60	42	7.98	27	5.31	24	4.56	7	1.32	19	3.54

*Enterococcus *CFU^2^												
Non-swimmer	159	5.86	100	3.69	109	4.07	68	2.49	35	1.26	86	3.07

0.606,2.32	43	7.39	27	4.64	29	5.06	26	4.48	15	2.51	17	2.82

2.32,4.29	29	6.92	19	4.55	20	4.78	15	3.55	9	2.12	8	1.86

4.29,10.2	35	7.64	23	5.02	23	5.13	22	4.73	5	1.06	15	3.16

10.2,22.9	46	7.36	30	4.80	36	5.85	21	3.32	7	1.10	15	2.33

22.9,230	54	11.46	37	7.86	35	7.59	16	3.36	11	2.32	17	3.56

Fecal *Bacteroides *${\text{CCE}}_{\Delta \Delta }^{1}$												
Non-swimmer	159	5.86	100	3.69	109	4.07	68	2.49	35	1.26	86	3.07

26.2,69	22	5.63	14	3.58	18	4.68	12	3.01	8	1.98	8	1.99

69,224	25	6.11	16	3.91	31	7.64	20	4.82	6	1.44	15	3.57

224,802	33	9.97	22	6.65	20	6.08	15	4.45	5	1.46	5	1.45

802,2.06e+03	30	8.26	22	6.06	24	6.63	10	2.73	9	2.44	15	4.02

2.06e+03,6.08e+03	28	8.07	18	5.19	13	3.88	11	3.20	8	2.29	11	3.12

*Clostridium *${\text{CCE}}_{\Delta \Delta }^{1}$												
Non-swimmer	159	5.86	100	3.69	109	4.07	68	2.49	35	1.26	86	3.07

157,503	22	5.47	16	3.98	25	6.27	21	5.11	8	1.92	13	3.10

503,890	24	6.90	17	4.89	15	4.36	14	4.00	2	0.57	13	3.68

890,1.45e+03	29	6.94	18	4.31	23	5.50	14	3.29	14	3.25	12	2.76

1.45e+03,1.79e+03	33	9.73	26	7.67	19	5.81	11	3.27	6	1.76	8	2.34

1.79e+03,3.51e+03	30	8.98	15	4.49	24	7.29	8	2.37	6	1.76	8	2.33

1: Calibrator Cell Equivalents per 100 ml calculated by delta delta-CT (CCE_ΔΔ_)

2: Colony Forming Units per 100 ml

GI: gastrointestinal illness, URI: Upper respiratory illness

**Figure 2:**
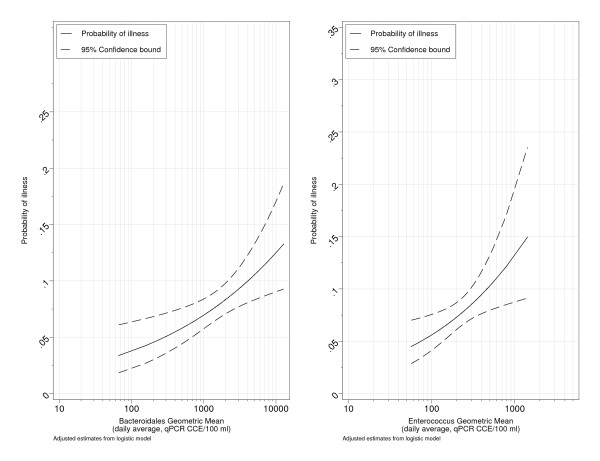
**Estimated probability of GI illness among swimmers as a function of daily averages of Enterococcus and Bacteroidales qPCR CCE_ΔΔ_**.

**Figure 3:**
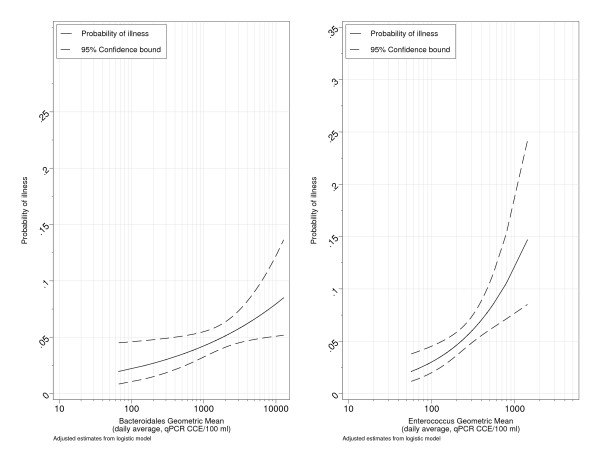
**Estimated probability of Diarrhea among swimmers as a function of daily averages of Enterococcus and Bacteroidales qPCR CCE_ΔΔ_**.

AORs of illness among swimmers with respect to indicator density are shown for all subjects (Table 4). The risks of both GI illness and diarrhea were significantly associated with exposure to *Enterococcus *and *Bacteroidales *CCE_Δ _and CCE_ΔΔ _(Figures 2 and 3). A log_10 _increase in the daily average of *Enterococcus *and *Bacteroidales *CCE was associated with an approximate doubling or greater (AORs≈2 or more) in the risk of GI illness (Table 4). Morning estimates of *Bacteroidales *and *Enterococcus *CCE (8:00 and/or 11:00 AM) were also significantly associated with both GI illness and diarrhea. Associations between GI illness and fecal *Bacteroidales *were generally positive but not statistically significant. *Clostridium *CCE_ΔΔ _showed a strong statistically significant association with GI illness (AOR = 1.94, 95% CI 1.16-3.23, Table 4), but the relationship with Clostridium CCE_Δ _was weaker (AOR = 1.81, 95% CI 0.96-3.39), Table 4).

**Table 4:** Adjusted Odds Ratios for illness risk among swimmers for a 1 log_10 _increase in indicator density^1^

	GI		Diarrhea		URI	
			
	AOR	95% CI	AOR	95% CI	AOR	95% CI
*Enterococcus *CFU^2^						

8:00 AM	1.20	0.91-1.59	1.33	0.92-1.91	1.04	0.73-1.46

11:00 AM	1.16	0.92-1.45	1.19	0.89-1.6	1.13	0.88-1.46

Daily	1.16	0.84-1.61	1.22	0.81-1.86	1.11	0.75-1.63

*Enterococcus *${\text{CCE}}_{\Delta }^{3}$						

8:00 AM	1.94	1-3.8	2.57	1.15-5.73	1.46	0.65-3.29

11:00 AM	2.08	1.18-3.67	2.54	1.29-4.99	1.62	0.86-3.04

Daily	2.67	1.34-5.34	3.57	1.57-8.13	1.83	0.76-4.39

*Bacteroidales *${\text{CCE}}_{\Delta }^{3}$						

8:00 AM	1.61	1.21-2.16	1.69	1.11-2.58	1.13	0.84-1.52

11:00 AM	1.43	1.02-1.99	1.40	0.92-2.14	0.93	0.67-1.29

Daily	1.67	1.16-2.39	1.68	1.08-2.61	0.96	0.66-1.38

*Fecal Bacteroides *${\text{CCE}}_{\Delta }^{3}$						

8:00 AM	1.30	0.99-1.69	1.34	0.94-1.91	1.12	0.81-1.54

11:00 AM	1.15	0.91-1.45	1.14	0.84-1.54	0.93	0.72-1.2

Daily	1.27	0.98-1.64	1.26	0.9-1.75	0.95	0.69-1.31

*Clostridia *sp. ${\text{CCE}}_{\Delta }^{3}$						

8:00 AM	1.81	0.96-3.39	1.43	0.67-3.08	1.05	0.56-1.95

11:00 AM	1.32	0.68-2.56	1.46	0.6-3.55	0.93	0.54-1.61

Daily	1.87	0.75-4.67	1.68	0.56-5.05	1.24	0.49-3.16

*Enterococcus *${\text{CCE}}_{\Delta \Delta }^{3}$						

8:00 AM	1.57	0.83-2.94	2.33	1.06-5.12	1.45	0.69-3.02

11:00 AM	2.11	1.27-3.52	2.97	1.63-5.42	1.57	0.79-3.11

Daily	2.56	1.29-5.11	4.42	1.96-9.94	1.88	0.79-4.5

*Bacteroidales *${\text{CCE}}_{\Delta \Delta }^{3}$						

8:00 AM	1.71	1.23-2.36	1.87	1.16-3	1.13	0.82-1.55

11:00 AM	1.51	1.05-2.17	1.56	0.97-2.5	0.86	0.6-1.22

Daily	1.91	1.26-2.9	1.96	1.11-3.47	0.88	0.59-1.31

Fecal *Bacteroides *${\text{CCE}}_{\Delta \Delta }^{3}$						

8:00 AM	1.29	0.96-1.74	1.38	0.92-2.07	1.15	0.83-1.61

11:00 AM	1.14	0.88-1.48	1.14	0.82-1.6	0.93	0.71-1.22

Daily	1.26	0.94-1.68	1.26	0.86-1.86	0.96	0.69-1.33

*Clostridia *sp. ${\text{CCE}}_{\Delta \Delta }^{3}$						

8:00 AM	1.94	1.16-3.23	1.62	0.86-3.07	0.99	0.56-1.74

11:00 AM	1.97	1.03-3.77	2.21	0.96-5.13	0.82	0.46-1.46

Daily	2.76	1.29-5.89	2.45	0.96-6.29	1.05	0.47-2.32

1: Exposure indices assigned for daily average of all samples, average of 8:00 AM samples and average of 11:00 AM samples

2: log_10 _colony forming units per 100 ml

3: log_10 _qPCR Calibrator Cell Equivalents per 100 ml

GI: gastrointestinal illness, URI: Upper respiratory illness, AOR: Adjusted Odds Ratio, 95% CI: 95% Confidence Interval

*Enterococcus *CFU were also positively associated with GI illness, but the associations were not statistically significant. None of the associations with non-enteric illnesses were consistently positive (data for rash, earache and eye irritations see additional file 2, Table S1).

Children 10 years of age and under were examined separately (see additional file 2, Table S2) but there was no evidence of an increased susceptibility to illness with exposure to fecal indicator bacteria. Statistical models which allowed a separate slope for children showed no improvement over models with a single slope for all subjects.

Associations between GI illness and qPCR CCE appeared to strengthen with more intense or prolonged water exposure, although results were based on few subjects. For example, among those exposed to water greater than 90 minutes (N = 751), AORs were 6.4 (95% CI: 1.2-33) and 7.14 (95% CI: 1.4-37) for associations between Enterococcus CCE_ΔΔ _and GI illness and diarrhea, respectively. Among subjects who swallowed water (N = 632) the AOR for GI illness with respect to the daily average *Enterococcus *CCE_ΔΔ _was 8.9 (95% CI: 2.2-37).

Associations between GI illness and qPCR CCE were robust to many ways of handling and interpreting the data. Allowing qPCR CT values to extend to either 40 or 45 for CCE calculations and using different approaches to impute results for non-detected samples [31] such as a maximum likelihood, regression on order statistics, or Kaplan-Meier estimates did not affect the results or the interpretation (data not shown). Excluding those with swimming contact in the previous 1-week reduced the sample size, but also had little affect on the results (for example: AOR = 2.4, 95% CI 1.04-5.5 and AOR = 2.3, 95% CI 1.3-3.8 for associations between GI illness and *Enterococcus *and *Bacteroidales *CCE_ΔΔ_, respectively). Restricting the definition of swimmers to those who immersed their head also had little effect on the results as did using averages of water quality approximately specific to the swimmer's estimated time of day and location of swimming exposure (data not shown).

There was no evidence of systematic differences in the relationships between GI illness and diarrhea and *Enterococcus *or *Bacteroidales *CCE exposure across the three beaches. Likelihood ratio tests comparing a full model which allowed the slope to vary by beach was no better than a model that allowed a common slope (for example: p = 0.51 and 0.90 for GI illness associations with *Enterococcus *and *Bacteroidales *CCE_ΔΔ_, respectively).

#### F+ Coliphage and Illness

The odds of GI illness was higher among swimmers compared to non-swimmers on days when F+ DNA or F+ RNA coliphage was detected by the CLAT assay or F+ coliphage was detected by the SPOT assay (Table 5). Positive associations between GI illness and F+ RNA and F+ DNA coliphage measured by the CLAT assay among swimmers were not statistically significant (Table 5), although statistical power was limited due to relatively few positive results. F+ coliphage measured by the SPOT assay was also not associated with GI illness among swimmers (Table 5). Other illnesses did not show a relationship with the presence of coliphage (data not shown).

**Table 5:** GI illness by swimming status and presence of F+ coliphage by 24-hour SPOT Assay and 5-hour CLAT Assay

	Number ill	%	**AOR**^**2**^**(95% CI)**	**AOR-trend**^**3**^**(95% CI)**
**F+ Coliphage Spot Assay**^1^				
Non-swimmer	87	4.90	ref	

0.1, 0.7	39	6.74	1.52(0.98-2.36)	

0.7, 24	64	8.47	1.699(1.12-2.57)	1.15(0.69-1.92)

**F+ RNA Coliphage CLAT Assay**				
Non-swimmer	87	4.90	ref	

Absent^4^	39	6.69	1.18(0.74-1.88)	

Present^4^	64	8.51	1.8(1.22-2.66)	1.55(0.9-2.66)

**F+ DNA Coliphage CLAT Assay**				
Non-swimmer	87	4.90	ref	

Absent^4^	23	6.05	1.11(0.64-1.93)	

Present^4^	80	8.38	1.69(1.16-2.47)	1.61(0.86-3)

1: Daily Geometric Mean, Plaque forming units per 100 ml estimated by Most Probable Number (MPN) Method

2: Adjusted Odds Ratio comparing exposed swimmers to non-swimming reference group

3: Adjusted Odds Ratio for a 1-log increase in coliphage on a continuous scale among swimmers

4: Swimmers exposed on days when coliphage absent or present by the CLAT assay

#### Illness below and above current indicator criteria

Eleven of the 70 days studied exceeded the *Enterococcus *CFU guideline geometric mean value of 35 CFU per 100. On these days, we observed elevated odds of diarrhea, respiratory illness and earache among swimmers compared to non-swimmers (Table 6). For all categories of illness, there were no differences in the odds of illness among swimmers on days when *Enterococcus *exceeded 35 CFU/100 ml compared to days when *Enterococcus *was below 35 CFU/100 ml (Table 6).

**Table 6:** Illness by exposure to *Enterococcus *CFU, above and below EPA guidelines

	Number ill	%	**AOR**^**1 **^**(95% CI)**	**AOR**^**2 **^**(95% CI)**
**GI**				
Non-swimmer	159	5.86	ref	

Swimmer-below 35 CFU	171	7.7		ref

Swimmer-above 35 CFU	36	10.75	1.52(0.96-2.4)	1.2(0.75-1.91)

Total	366	6.94		

**Diarrhea**				
Non-swimmer	100	3.69	ref	

Swimmer-below 35 CFU	108	4.87		ref

Swimmer-above 35 CFU	28	8.36	1.78(1.02-3.09)	1.4(0.8-2.43)

Total	236	4.48		

**URI**				
Non-swimmer	109	4.07		

Swimmer-below 35 CFU	116	5.3		ref

Swimmer-above 35 CFU	27	8.28	1.78(1.02-3.11)	1.35(0.77-2.38)

Total	252	4.85		

**Rash**				
Non-swimmer	68	2.49	ref	

Swimmer-below 35 CFU	92	4.11		ref

Swimmer-above 35 CFU	8	2.36	1.12(0.47-2.68)	0.68(0.28-1.63)

Total	168	3.17		

**Earache**				
Non-swimmer	35	1.26	ref	

Swimmer-below 35 CFU	37	1.63		ref

Swimmer-above 35 CFU	10	2.93	2.37(1.06-5.31)	1.8(0.84-3.83)

Total	82	1.53		

**Eye irritation**				
Non-swimmer	86	3.07	ref	

Swimmer-below 35 CFU	60	2.62		ref

Swimmer-above 35 CFU	12	3.53	1.09(0.56-2.15)	1.3(0.65-2.59)

Total	158	2.91		

1: Adjusted Odds Ratio: Swimmers Above 35 CFU vs. non-swimmers

2: Adjusted Odds Ratio: Swimmers above 35 CFU vs. swimmers below 35 CFU

GI: Gastrointestinal illness; URI: Upper Respiratory Illness; 95% CI: 95% Confidence interval

## Discussion

This is the first study to demonstrate a relationship between rapid, molecular measures of fecal indicator organisms and swimming-associated illness at marine beaches. As we observed at freshwater beaches, of the various symptom categories those most consistently associated with fecal indicator bacteria were symptoms of GI illness. Although daily averaged samples generally showed the strongest associations with illness, the six morning samples collected at either 8:00 AM or 11:00 AM also were associated with illness occurrence.

The different CCE calculation approaches had little effect on the interpretation of the results. The finding that associations were unaffected by the type of CCE calculation and that the associations strengthened among those with increased exposure (swallowing water, and among those exposure longer than 90 minutes) reinforced the validity of the results.

Average water quality as measured by *Enterococcus *CFU at these three marine beaches was relatively good. Most other epidemiology studies at marine beaches have reported higher levels of *Enterococcus *CFU (although direct comparisons are difficult due to differences in the way average indicator densities were reported) [32-34]. A series of randomized studies in the United Kingdom, however, observed exposure-response associations at marine beaches impacted by sewage with low levels of *Enterococcus *CFU exposures [35]. Here, we report an association between *Enteroccocus *and *Bacteroidales *CCE and GI illness despite relatively low levels of culturable *Enterococcus*. This is consistent with our previous findings at freshwater beaches and provides further evidence that fecal indicators estimated by qPCR are a sensitive marker of poor water quality and subsequent health effects among swimmers at beach sites impacted by treated sewage discharge. Compared to the freshwater beach sites we previously studied [6], average *Enterococcus *CCE were slightly higher and average *Enterococcus *CFU were lower. The relationship between *Enterococcus *qPCR CCE and *Enterococcus *CFU can be influenced by many factors and may vary from beach to beach [36]. Environmental factors, such as sunlight, may affect the differential persistence of the qPCR signal and culturable organisms [37-39].

The estimate of the association between *Enterococcus *CCE_ΔΔ _and GI illness among swimmers (AOR = 2.59, 95%CI 1.29-5.11) was stronger than we reported at freshwater beaches (AOR = 1.26, 95% CI 95% CI 1.05-1.51) [6]. Although the 95% confidence bounds of the AORs for marine and freshwater overlap, such an overlap does not necessarily indicate no statistical difference [40,41]. Our sample size at the marine beaches was smaller and as a result, the 95% confidence bounds are wider and the AOR is less precise. Previous studies [8] also observed a higher illness rate and slope for the association between culturable *Enterococcus *and GI illness in marine water which the authors suggested may have been due to a greater die off of indicators relative to pathogens in marine waters. We anticipate more detailed comparisons of these results will be the focus of future reports which should also consider baseline (non-swimmer) illness. We observed positive relationships between gastrointestinal symptoms and other fecal indicators, *Clostridium*, fecal *Bacteroides *CCE, as well as F+ coliphage, but since associations were not consistently statistically significant among swimmers we cannot make conclusions regarding these indicators.

*Clostridium perfringens *has been suggested for use as an indicator of fecal contamination in tropical environments [42], but it has not been previously associated with illness in epidemiological studies [3]. Male-specific coliphage also showed some evidence of an association with GI illness at a marine site in Mission Bay, California [32] but this was based on few positive samples. Levels of F+ RNA coliphage were associated with gastrointestinal illnesses among canoeists in freshwater rivers [43].

### Limitations

This study was conducted at beaches in a temperate climate with nearby treated point source sewage discharges and results may not be directly applicable to sites affected by fecal contamination from other types of sources or sites with different climates. Human viruses such as norovirus have been identified as likely contributors to excess GI illness among swimmers at beaches impacted by treated sewage discharges [44]. Some human pathogens, particularly human enteric viruses, are unlikely to be associated with non-human sources of fecal indicator bacteria. Results from some recent studies support this limitation. A recently conducted randomized study in Flordia at a sub-tropical beach without known sources of sewage showed no association between fecal indicator bacteria levels and GI illness (including *Enterococcus*, and three *Bacteroidales *markers measured by qPCR [34]). This study did observe an association between skin rash and culturable *Enterococcus *[34,45], which they suggested may have been marking conditions favorable to pathogens which could cause skin infections [34]. A study at a California marine beach site also failed to find any robust associations between fecal indicator organisms and swimming-associated illness, including *Enterococcus *and *Bacteroidales *measured by qPCR [32]. This study, conducted at a beach that was impacted by widespread and diffuse sources of fecal contamination but no point source of contamination [46]. Source tracking studies identified birds as the predominant source of fecal pollution [47]. The results we report from these temperate marine beach sites also may not be applicable to a humid tropical environmental where fecal indicator bacteria have been reported to persist or accummulate in the soil [48].

We used an observational cohort study design which has been used by numerous others both historically and recently to evaluate the risks associated with recreational water exposures (for example [32,33,49-52]). This design allowed us to establish associations between average water quality and the subsequent risk of illness among those exposed. One disadvantage of this approach is that such measures of average water quality may not represent an individual swimmer's true exposure. However, in our study, swimmers were exposed for long durations (an average of 74 minutes, with 25% exposed more than 2 hours) and entered the water at multiple time periods and locations, making attempts to characterize individual exposure difficult and impractical. This prolonged and discontinuous exposure may explain why attempts to create time and location specific averages of water quality measures for each swimmer showed no advantage over broader characterizations of water quality. An advantage of the study design we used is the ability to field the study over a wide range of study days which resulted in varying average water quality conditions and enrollment of a large study population. Furthermore, our sampling design allowed us to demonstrate that estimates *Enterococcus *and *Bacteroidales *CCE in six morning samples were associated with subsequent GI illness among swimmers exposed on that day.

Some of the health endpoints were non-specific, and may have been affected by recall bias. Broad endpoints accounted for the diverse range of symptoms potentially associated with recreational water exposure but such broad symptoms may obscure more specific effects of water quality and swimming exposure. The associations *Enterococcus *and *Bacteroidales *CCE and gastrointestinal symptoms, however, were robust to different definitions (e.g., diarrhea). While swimmers may have been more likely to recall illness than non-swimmers, it is unlikely such a recall bias would occur among swimmers at varying levels of water quality.

## Conclusion

Rapid, molecular measures of water quality were associated with illnesses among swimmers at marine beach sites located in a temperate climate with nearby treated sewage discharges. The results provide further evidence that such indicators are a marker of GI illness risk at such beach sites.

## List of Abbreviations

QPCR: Quantitative polymerase chain reaction; AOR: Adjusted odds ratio; P: P-value; CI: Confidence interval; GI: Gastrointestinal; URI: Upper respiratory illness; POTW: Publicly owned treatment works; CCE: Calibrator cell equivalents; CCE_ΔΔ_: Calibrator cell equivalents, delta-delta cycle threshold calculation; CCE_Δ_: Calibrator cell equivalents, delta cycle threshold calculation; CT: Cycle Threshold; CFU: Colony forming units; CLAT: Culture and latex agglutination assay for F+ coliphage; SPOT: 24-hour spot test for F+ coliphage.

## Competing interests

The authors declare that they have no competing interests.

## Authors' contributions

TW led study planning and implementation, data analysis, result interpretation and manuscript preparation. ES led survey and environmental data collection. KB led water sampling design and microbiological analysis. CR conducted microbiological analyses and coliphage testing. EC and RH conducted qPCR analysis and interpreted qPCR results. DL provided expertise on the rapid coliphage CLAT assay. QL conducted and provided input on statistical analyses. RN provided expertise on the Fecal *Bacteroides *assay. MB conceptualized the study, designed questionnaires and developed study materials. AD conceptualized the study, selected beach sites, and interpreted the results. All authors contributed to writing the manuscript and all authors read and approved the final manuscript.

## Supplementary Material

Additional file 1 **Supplemental information on qPCR assay and CCE calculations**. Click here for file

Additional file 2 **Additional tables**. Click here for file
