# Supplementary material for: Rapidly measured indicators of recreational water quality and swimming-associated illness at marine beaches: a prospective cohort study
Source: Environ Health. 2010 Oct 31;9:66. doi: 10.1186/1476-069X-9-66 (PMC2990738; doi:10.1186/1476-069X-9-66)
Supplement: Additional file 1 — Supplemental information on qPCR assay and CCE calculations. [file 1476-069X-9-66-S1.PDF]

qPCR sample processing, DNA extraction and calibrator standards

Details of sample processing and the DNA extraction procedure have been previously described [1, 2]. Fifty milliliters of water samples, collected from Edgewater and Fairhope Beach and 100 mL from Goddard Beach were filtered through a 0.4 μm pore size (47 mm in diameter) polycarbonate membrane filter (GE Osmonics, Minnetonka, MA), and the sides of the funnels were rinsed twice with 20 ml of sterile, PCR-grade water. Using sterile forceps, each filter was folded into a cylinder with the sample side facing inward, and then inserted into a 2 ml semiconical screwcap microcentrifuge tube (extraction tube) (catalog #506-636, PGC Scientific, Gaithersburg, MD) containing 0.3 g of acid-washed glass beads (catalog #G-1277, Sigma, St. Louis, MO). The filters were held at -20°Cuntil overnight shipment to EMSL Analytical (Westmount NJ) on dry ice where DNA extraction and qPCR analysis for *Enterococcus*, *Bacteroidales* and fecal *Bacteroides* were conducted as described below. Frozen DNA extracts were sent from EMSL Analytical to the US EPA in Cincinnati where qPCR analyses were conducted for *Clostridium* spp.

DNA was recovered from the organisms retained on the filters by addition of 600 μl of AE buffer (Qiagen, Valencia, CA) containing 0.2 $\mu g/ml$ salmon testes DNA (#D-1626, Sigma, St. Louis, MO), added as an exogenous, internal positive control and reference, to each extraction tube and bead milling in an eight position mini bead beater (Biospec Corp., Bartlesville, OK) for 60 s at maximum rate. The tubes were then centrifuged at 12,000 x g for 1 min to pellet the glass beads and debris. Resulting supernatants were transferred to sterile 1.6 ml low retention microcentrifuge tubes (GENE MATE, #C-3228-1) and, if not analyzed immediately, stored at -20 °C. Negative controls consisted of two filtrates of 40 ml PCR-grade water, prepared at the same time as the sample filtrates, and six blank filters prepared in the PCR analytical laboratory, that were extracted in the same manner with each batch of samples arriving weekly at the laboratory.

Calibrator samples (six replicates), consisting of clean polycarbonate filters amended with known cell quantities of Enterococcus faecalis (ATCC# 29212), Bacteroides thetaiotaomicron (ATCC# 29741), and Clostridium perfringens (ATCC# 13124)and negative control samples (six replicates), consisting of clean filters only, were extracted in the same manner with each batch of test samples. Cells used in the calibrator samples originated from laboratory grown cultures and were enumerated as previously described [2, 3, 1].

Following DNA extraction, polymerase chain reaction (PCR) amplification was carried out on 5-fold dilutions of the DNA extracts in AE buffer using the TaqMan PCR product detection system. The reactions were performed in a thermal cycling instrument (Smart-Cycler System, Cepheid, Sunnyvale, CA) except for *Clostridium* spp. which was performed on a Model 7900 DNA thermal cycler (Applied Biosystems, Foster City, CA). Both instruments automated the detection and quantitative measurement of the fluorescent signals

produced by TaqMan probe degradation during each cycle of amplification. Additional details regarding qPCR analysis including reaction conditions, reagent mixes and development calibrator standards are detailed in Haugland [2] and for *Clostridium* in Chern [1].

qPCR CCE calculations

The delta-delta CT ($CCE_{\Delta\Delta}$)computational approach is derived from the comparative cycle threshold (CT) method [4]. This approach employs an arithmetic formula to determine the ratio of target sequence quantities in DNA extracts from test sample filters relative to those in similarly-prepared DNA extracts from calibrator sample filters containing a known quantity of target organism cells based on the difference in CT values obtained from qPCR analyses of these samples. In the present application of the comparative CT method, the test samples were the water sample filters and the calibrator samples were filters containing a known quantity of target organism cells as described above. Similar comparisons of CT values from qPCR assays for an exogenous target sequence from salmon sperm DNA, added in equal quantities to both the test and calibrator sample filters before DNA extraction, were used both as a reference to normalize results for differences in the amount of total DNA recovered from each sample (e.g., caused by test sample effects on DNA recovery) and as a sample processing control (SPC) to signal potentially non-quantifiable test sample results caused by PCR inhibition or low DNA recoveries[2]. The calculation can be expressed by the following equations:

$$CT_{\Delta,\Delta} = \Delta C_{T,target} - \Delta C_{T,ref} \tag{1}$$

and

$$CCE_{\Delta,\Delta} = N_{calibrator} \times A^{-CT_{\Delta,\Delta}} \tag{2}$$

where:

- $\Delta C_{T,target}$ is the difference between the CT from the sample target (e.g., *Enterococcus* and the average CT of the batch calibrator
- $\Delta C_{T,ref}$ is the corresponding difference for the salmon sperm reference sequence
- $N_{calibrator}$ is the known number of cells in the calibrator sample
- A is the amplification factor for the assay.

Ideally A=2 but typically it is in the range 1.9 2.0 with values less than 2 resulting from less than 100% replication of the target sequence at each cycle. In practice, A is either assumed to be 2 or is estimated based on the slope of a standard curve[4]. For the *Enterococcus*, *Clostridium*, and *Bacteroidales* assays, values for A were assumed to be 2 because this value was within the

95% confidence intervals of the slope values obtained by the laboratory from repeated qPCR analyses of serially diluted genomic DNA standards. A was 1.94 for fecal *Bacteroidaes* and 1.89 for *Clostridium*.

For the delta-CT (CCE_{Δ}) calculation, the $\Delta C_{T,ref}$ above is excluded from the calculation and the Salmon assay is only used as a pass-fail control.

References

[1] Chern EC, Brenner KP, Wymer L, Haugland RA: **Comparison of Fecal Indicator Bacteria Densities in Marine Recreational Waters by QPCR**. *Water Quality Exposure and Health* 2009, **1**:203–214.

[2] Haugland RA, Siefring SC, Wymer LJ, Brenner KP, Dufour AP: **Comparison of Enterococcus measurements in freshwater at two recreational beaches by quantitative polymerase chain reaction and membrane filter culture analysis**. *Water Res* 2005, **39**(4):559–568.

[3] Siefring S, Varma M, Atikovic E, Wymer L, Haugland RA: **Improved real-time PCR assays for the detection of fecal indicator bacteria in surface waters with different instrument and reagent systems**. *Journal of Water Health* 2008, **6**(2):225–237.

[4] Applied Biosystems: **User Bulletin 2: ABI PRISM 7700 Sequence Detection System**. Tech. rep., Applied Biosystems Corporation 1997.
