# Supplementary material for: Rapidly measured indicators of recreational water quality and swimming-associated illness at marine beaches: a prospective cohort study
Source: Environ Health. 2010 Oct 31;9:66. doi: 10.1186/1476-069X-9-66 (PMC2990738; doi:10.1186/1476-069X-9-66)
Supplement: Additional file 2 — Additional tables. [file 1476-069X-9-66-S2.PDF]

Table S1: Adjusted Odds Ratios for illness risk among swimmers for a 1 $\log_{10}$ increase in indicator density[1]

	Rash		Earache		Eye	
	AOR	95% CI	AOR	95% CI	AOR	95% CI
Enterococcus CFU[2]						
8:00 AM	1.26	0.89-1.79	0.64	0.4-1.04	1.04	0.69-1.56
11:00 AM	0.85	0.6-1.21	0.68	0.46-1.01	1.17	0.83-1.66
Daily	0.94	0.59-1.48	0.69	0.38-1.23	1.24	0.78-1.97
Enterococcus CCE^3_{Δ}						
8:00 AM	1.40	0.6-3.25	2.07	0.81-5.29	1.47	0.53-4.08
11:00 AM	0.53	0.21-1.33	0.84	0.29-2.41	1.44	0.6-3.42
Daily	0.79	0.26-2.37	1.82	0.55-5.99	1.48	0.47-4.66
Bacteroidales CCE^3_{Δ}						
8:00 AM	1.02	0.75-1.4	1.06	0.68-1.67	1.35	0.96-1.89
11:00 AM	0.88	0.58-1.31	0.90	0.54-1.51	1.21	0.79-1.85
Daily	0.94	0.62-1.44	1.07	0.58-1.99	1.24	0.8-1.9
Fecal Bacteroides CCE^3_{Δ}						
8:00 AM	0.69	0.5-0.95	1.57	0.92-2.68	1.05	0.66-1.68
11:00 AM	0.68	0.49-0.95	1.32	0.86-2.03	0.95	0.65-1.37
Daily	0.69	0.48-1	1.45	0.86-2.44	0.99	0.64-1.53
Clostridia sp. CCE^3_{Δ}						
8:00 AM	0.51	0.25-1.06	0.84	0.26-2.68	1.19	0.57-2.49
11:00 AM	0.61	0.32-1.16	1.06	0.4-2.82	0.90	0.4-1.98
Daily	0.34	0.12-0.97	1.80	0.35-9.17	0.88	0.31-2.54
Enterococcus $CCE^3_{\Delta\Delta}$						
8:00 AM	1.47	0.56-3.84	1.15	0.41-3.21	1.04	0.39-2.75
11:00 AM	0.54	0.23-1.29	0.54	0.16-1.84	1.23	0.52-2.93
Daily	0.84	0.26-2.64	1.01	0.26-3.96	1.07	0.35-3.28
Bacteroidales $CCE^3_{\Delta\Delta}$						
8:00 AM	1.01	0.72-1.41	0.93	0.58-1.49	1.36	0.92-2
11:00 AM	0.80	0.51-1.24	0.74	0.43-1.26	1.18	0.74-1.9
Daily	0.86	0.52-1.42	0.89	0.47-1.69	1.24	0.75-2.07
Fecal *Bacteroides* $CCE^3_{\Delta\Delta}$						
8:00 AM	0.70	0.49-0.98	1.54	0.87-2.73	1.01	0.6-1.68
11:00 AM	0.72	0.49-1.04	1.26	0.8-1.98	0.92	0.63-1.34
Daily	0.72	0.47-1.1	1.41	0.83-2.38	0.95	0.6-1.5
Clostridia sp. $CCE^3_{\Delta\Delta}$						
8:00 AM	0.72	0.37-1.4	0.75	0.3-1.83	1.22	0.61-2.45
11:00 AM	0.71	0.34-1.49	0.70	0.3-1.64	1.02	0.45-2.33
Daily	0.49	0.21-1.14	1.17	0.3-4.63	1.07	0.38-3.03

1: Exposure indices assigned for daily average of all samples, average of 8:00 AM samples and average of 11:00 AM samples

2: $\log_{10}$ colony forming units per 100 ml

3: $\log_{10}$ qPCR Calibrator Cell Equivalents per 100 ml

GI: gastrointestinal illness, URI: Upper respiratory illness, AOR: Adjusted Odds Ratio, 95% CI: 95% Confidence Interval

Table S2: Adjusted Odds Ratios for illness risk among swimmers for a 1 $\log_{10}$ increase in indicator density[1]. Children age 10 and under.

	GI		Diarrhea		URI	
	AOR	95% CI	AOR	95% CI	AOR	95% CI
Enterococcus CFU[2]	0.97	0.54-1.75	1.30	0.62-2.72	1.25	0.68-2.29
Enterococcus CCE^3_{Δ}	2.69	0.93-7.77	4.04	1.17-13.98	3.42	1.14-10.19
Bacteroidales CCE^3_{Δ}	2.15	1.29-3.57	2.22	1.11-4.45	1.04	0.6-1.81
Fecal *Bacteroides* CCE^3_{Δ}	1.42	0.96-2.1	1.63	0.94-2.83	1.31	0.85-2.01
Clostridium spp. CCE^3_{Δ}	3.01	0.72-12.61	2.56	0.48-13.8	1.19	0.31-4.53
Enterococcus $CCE^3_{\Delta\Delta}$	2.04	0.7-5.96	3.55	0.91-13.9	2.99	0.82-10.87
Bacteroidales $CCE^3_{\Delta\Delta}$	2.35	1.32-4.19	2.38	1.02-5.55	0.95	0.51-1.77
Fecal *Bacteroides* $CCE^3_{\Delta\Delta}$	1.38	0.87-2.19	1.67	0.84-3.3	1.36	0.84-2.22
Clostridium spp. $CCE^3_{\Delta\Delta}$	4.68	1.02-21.4	3.70	0.48-28.46	0.92	0.29-2.95

1: Exposure indices assigned for daily average of all samples
2: $\log_{10}$ colony forming units per 100 ml
3: $\log_{10}$ qPCR calibrator cell equivalents per 100 ml
GI: gastrointestinal illness, URI: Upper respiratory illness, AOR: Adjusted Odds Ratio, 95% CI: 95% Confidence Interval
